# Development and validation of a dynamic risk prediction system for constipation in patients with amyotrophic lateral sclerosis

**DOI:** 10.3389/fneur.2022.1060715

**Published:** 2022-12-07

**Authors:** Tongyang Niu, Xiaomeng Zhou, Xin Li, Tingting Liu, Qi Liu, Rui Li, Yaling Liu, Hui Dong

**Affiliations:** ^1^Department of Neurology, Second Hospital of Hebei Medical University, Shijiazhuang, Hebei, China; ^2^Key Laboratory of Neurology of Hebei Province, Shijiazhuang, China

**Keywords:** amyotrophic lateral sclerosis, constipation, dynamic nomogram, model validation, risk factors

## Abstract

**Introduction:**

Although constipation is a common non-motor symptom in patients with amyotrophic lateral sclerosis (ALS), it is poorly valued. Moreover, there is a bidirectional effect between constipation and neuropsychiatric and sleep disturbances. Thus, these symptoms are better treated simultaneously. Therefore, this study aimed to develop and validate a model for predicting the risk of constipation in ALS patients, to help clinicians identify and treat constipation early.

**Methods:**

Data of 118 ALS admissions from an observational prospective cohort, registered between March 2017 and December 2021, were analyzed. Demographic data were obtained. Constipation was assessed using the Knowles–Eccersley–Scott Symptom Questionnaire. The severity of ALS was assessed using the Amyotrophic Lateral Sclerosis Functional Rating Scale-Revised (ALSFRS-R). Anxiety and depressive symptoms were measured using the Hospital Anxiety and Depression Scale (HADS). The Pittsburgh Sleep Quality Index (PSQI) was used to assess patients' sleep status. The least absolute shrinkage and selection operator (LASSO) regression model was used to select factors and construct a nomogram. Nomogram model performance was evaluated using the area under the receiver operating characteristic curve (AUC), calibration curve, decision curve analysis (DCA), and clinical impact curve (CIC). The model was internally validated using bootstrap validation in the current cohort.

**Results:**

Age, family history of constipation, total ALSFRS-R score, site of onset, total PSQI score, and depressed, were identified as significant predictors of the risk of constipation in ALS patients. The prediction model was validated to have good accuracy (Hosmer–Lemeshow test: χ^2^ = 11.11, *P* > 0.05) and discrimination (AUC = 0.856, 95% confidence interval: 0.784–0.928). DCA and CIC showed that the nomogram model had excellent clinical performance.

**Conclusions:**

A web-based ALS constipation risk calculator with good predictive performance was constructed to identify patients at high risk of constipation and to allow early intervention in a clinical context.

## Introduction

Amyotrophic lateral sclerosis (ALS) is a rare neurodegenerative disease that is traditionally thought to affect primarily the motor system. It is characterized by progressive degeneration of upper and lower motor neurons, leading to continued deterioration, with random muscle paralysis ensuing until death occurs (1). However, there is growing evidence that ALS is a multisystem disease, with 80% of patients reporting at least one non-motor symptom (2), such as constipation, sleep disturbance, autonomic dysfunction, cognitive impairment, and salivation. The presence of many of these symptoms are associated with shorter survival times and reduced quality of life. As there is no effective treatment for ALS, symptomatic supportive therapy is currently the main focus, aiming to prolong patient survival and to improve quality of life.

Constipation is one of the more common non-motor symptoms of ALS. Nevertheless, to date, constipation-related studies in ALS have been scarce, suggesting that less attention is paid to constipation than to other non-motor symptoms, such as cognitive impairment, neuropsychiatric symptoms, salivation, and sleep disturbances. The incidence of constipation in ALS has previously been reported to range between 46.0% and 68.3% (3–6), which is much higher than the incidence in the general population (14.0%) (7). However, Cheng et al. (8) found a low level of self-awareness of idiopathic constipation in Asian populations: amongst the constipated patients, only 57.4% were aware of having constipation. Patients with ALS themselves also focus more on their muscle atrophy and weakness, and it is assumed that some patients are not aware of their constipation and are unable to indicate this as one of their symptoms to neurologists. Consequently, underdiagnosis of constipation symptoms in patients frequently occurs, and therefore non-motor symptoms cannot be fully assessed.

Chronic constipation not only causes focal harm but also systemic harm. Patients with constipation often have coexisting anxiety, depression, and sleep disorders (9). Moreover, there is a well-documented bidirectional link between chronic constipation and anxiety/depression and sleep disorders. Chronic constipation can affect the level of anxiety and depression as well as the quality of sleep, in addition to being an important risk factor for anxiety/depression and sleep disorders (10, 11). Furthermore, simultaneous treatment of all three symptoms is more effective than is single symptom treatment. Chronic constipation, anxiety and depression, and sleep disorders are highly prevalent among ALS patients, and the co-existence of several non-motor symptoms is also very common in clinical practice.

Taken together, constipation causes severe pain, aggravates the anxiety/depression state and sleep disorders, which affects quality of life and prognosis (12–14) of ALS patients. However, given that use of certain drugs can treat or relieve constipation, this symptom should receive more attention expecting to improve the quality of life of patients with ALS and to improve their outcomes.

To our knowledge, no studies have been conducted to develop a predictive model for the risk of constipation in patients with ALS. Therefore, the aim of this study was to develop and validate a dynamic nomogram to help clinicians provide personalized early intervention treatment for ALS patients.

## Materials and methods

### Patient selection

**Figure 1:**
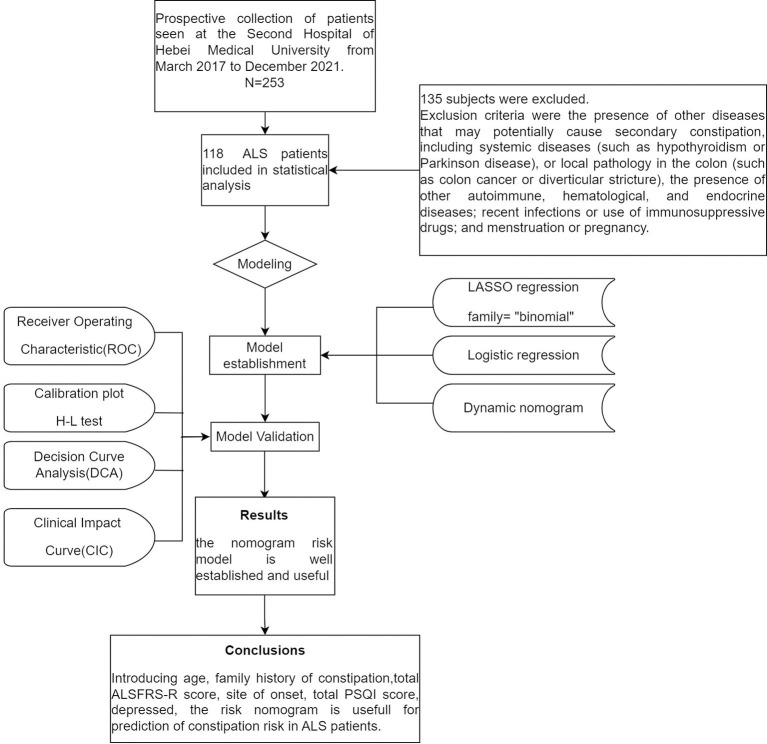
Flow diagram of study design.

This was an ancillary analysis of a prospective observational single-center study conducted over 57 months (from March 2017 to December 2021) at The Second Hospital of Hebei Medical University. Subjects diagnosed with clinically possible, clinically probable, or clinically definite ALS according to the El Escorial Criteria, and who were not taking medication that could affect bowel movements or defecation (e.g., sleeping pills, antispasmodics, opioids, etc.) were included. Exclusion criteria were the presence of other diseases that may potentially cause secondary constipation, including systemic diseases (such as hypothyroidism or Parkinson disease), or local pathology in the colon (such as colon cancer or diverticular stricture), the presence of other autoimmune, hematological, and endocrine diseases; recent infections or use of immunosuppressive drugs; and menstruation or pregnancy. Eventually, 118 patients were included in the study (Figure 1).

This study was approved by the Research Ethics Committee of the Second Hospital of Hebei Medical University (Shijiazhuang, Hebei, China; Approval No. R413) and complied with the code of ethics of the World Medical Association. We provided a complete explanation of the study protocol and obtained written informed consent from all participants prior to enrolling them in the study.

### Research methods

We reviewed the literature and guidelines on chronic constipation and collected previously reported factors affecting constipation (15–18). Based on clinical experience, these factors were summarized and investigated. Constipation was defined by the Rome III criteria for functional constipation (19). Accordingly, we divided patients into a constipation group and a non-constipation group.

We obtained basic patient information, including age, sex, duration of disease, obesity, dietary pattern, living environment, history of smoking and alcohol drinking, marital history, educational style, family history of constipation, etc. Additionally, we obtained information related to ALS constipation, including site of disease onset, disease course, anxiety and depression, and sleep status. We further assessed the prevalence and severity of constipation by using the Knowles–Eccersley–Scott Symptom Questionnaire (KESS) (20). The severity of ALS was assessed by the Amyotrophic Lateral Sclerosis Functional Rating Scale-Revised (ALSFRS-R). Anxiety and depressive symptoms were measured by the Hospital Anxiety and Depression Scale (HADS). A score on the HADS-anxiety or -depression subscales >7 indicated the presence of anxiety or depressive states, respectively, while scores ≤ 7 were considered normal (21). The Pittsburgh Sleep Quality Index (PSQI) was used to assess the sleep status of patients (22). Finally, in order to ensure that ALS patients maintain basic nutrition and water intake, we use the modified barium swallow (MBS) to evaluate whether there it exists unsafe swallowing, if exists, we consider percutaneous endoscopic gastrostomy (PEG), or nasogastric tube (NG) feeding if no other procedure is possible (23, 24).

### Statistical analysis

All data were statistically analyzed using SPSS v26.0 (IBM Corp., Armonk, NY, USA). We performed multiple imputations using Bayesian methods to fill in the missing data. The independent *t*-test was used to assess the PSQI total score as it was the only normally distributed variable. For other variables, the Mann–Whitney *U*-test was used. Categorical variables are presented as frequencies and percentages and were analyzed using Fisher's exact test. Continuous variables are reported as mean ± standard deviations if they are normally distributed or as median [interquartile range] for skewed distributions. Correlations were analyzed using Spearman's coefficient.

To optimize the calibration of the model and use less sample size to screen more variables efficiently, a penalized logistic regression model was estimated by the least absolute shrinkage and selection operator (LASSO) method for the variables included in the final model. LASSO shrinks data values toward a central point or mean and adds a penalty to the absolute value of the magnitude of the coefficients. Variable reduction was performed during LASSO regression by 10-fold cross-validation of the lambda value (25). The final model incorporated the variables that were most predictive within one standard error of the best value. This was followed by use of multivariate logistic regression analysis to construct predictive models from the variables selected in the LASSO regression model, with 95% confidence intervals (CI), odds ratios (ORs), and *P*-values. Finally, the results of the multivariate logistic regression analysis were visualized using a nomogram.

R software and its rms package (https://www.r-project.org/) were used to draw the nomogram. To facilitate its application in clinical practice, an interactive web-based dynamic nomogram application was built using the Shiny version. The predictive ability of the nomogram model was evaluated by using the receiver operating characteristic (ROC) (26) curve and area under the curve (AUC). ROC analysis was used to calculate the optimal cutoff value, determined by maximizing the Youden index. The accuracy of the cutoff value was assessed by calculating the sensitivity, specificity, and predictive values. The bootstrap method was used for internal validation: samples were randomly selected from the original data set and 1,000 repetitions were performed to assess the discrimination of the nomogram prediction model by calculating the mean consistency index (C-index), with values closer to 1 indicating better discrimination ability. The Hosmer–Lemeshow test, Brier score, and calibration curve were used to assess the calibration ability. Decision curve analysis (DCA) was used to determine the net-benefit threshold of prediction (27). Finally, a clinical impact curve (CIC) was plotted to assess the clinical utility and applicability of the model. Results with *P*-value < 0.05 were considered statistically significant.

## Results

### Single factor analysis affecting the occurrence of constipation in ALS patients

Based on the inclusion and exclusion criteria, 118 patients with ALS were recruited, including 77 males and 41 females. All patients were married. ALS is a hypermetabolic disease; thus, none of the patients were overweight (BMI < 30 kg/m^2^) (15). Consequently, marital and obesity factors were not included in the statistical analysis. According to the Rome III functional constipation criteria, constipation was present in 75 cases (63.6%). The study subjects were accordingly divided into a constipation group (*n* = 75) and a non-constipation group (*n* = 43). The general demographic characteristics and other information were compared between the two groups (Table 1).

**Table 1:** Comparison of demographic characteristics and general data of ALS patients.

**Characteristic**	**Non-constipation group (*n* = 43)**	**Constipation group (*n* = 75)**
Age	56.00 (17.00)	64.00 (17.00)
Sex	
Male	29 (67.4%)	48 (64.0%)
Female	14 (32.6%)	27 (36.0%)
Duration of disease	17.00 (18.00)	19.00 (23.00)
Family history of constipation	
Yes	1 (2.3%)	11 (14.7%)
No	42 (97.7%)	64 (85.3%)
Site of onset	
Bulbar	19 (44.2%)	24 (32.0%)
Limb	24 (55.8%)	51 (68.0%)
Diet	
General food	34 (79.1%)	47 (62.7%)
Liquid food	9 (20.9%)	28 (37.3%)
Alcohol	
Yes	10 (23.3%)	12 (16.0%)
No	33 (76.7%)	63 (84.0%)
Smoking	
Yes	12 (27.9%)	13 (17.3%)
No	31 (72.1%)	62 (82.7%)
Anxiety	
Yes	5 (11.6%)	9 (12.0%)
No	38 (88.4%)	66 (88.0%)
Depressed	
Yes	2 (4.7%)	24 (32.0%)
No	41 (95.3%)	51 (68.0%)
Living environment	
Rural	36 (83.7%)	49 (65.3%)
City	7 (16.3%)	26 (34.7%)
Nine year compulsory education	
Yes	11 (25.6%)	24 (32.0%)
No	32 (74.4%)	51 (68.0%)
Glycerine enema treatment	
Yes	NA	21 (28.0%)
No	NA	54 (72.0%)
ALSFRS-R total score	40.00 (12.00)	27.00 (19.00)
PSQI total score	6.12 ± 2.75	8.75 ± 3.72

### LASSO regression

**Figure 2:**
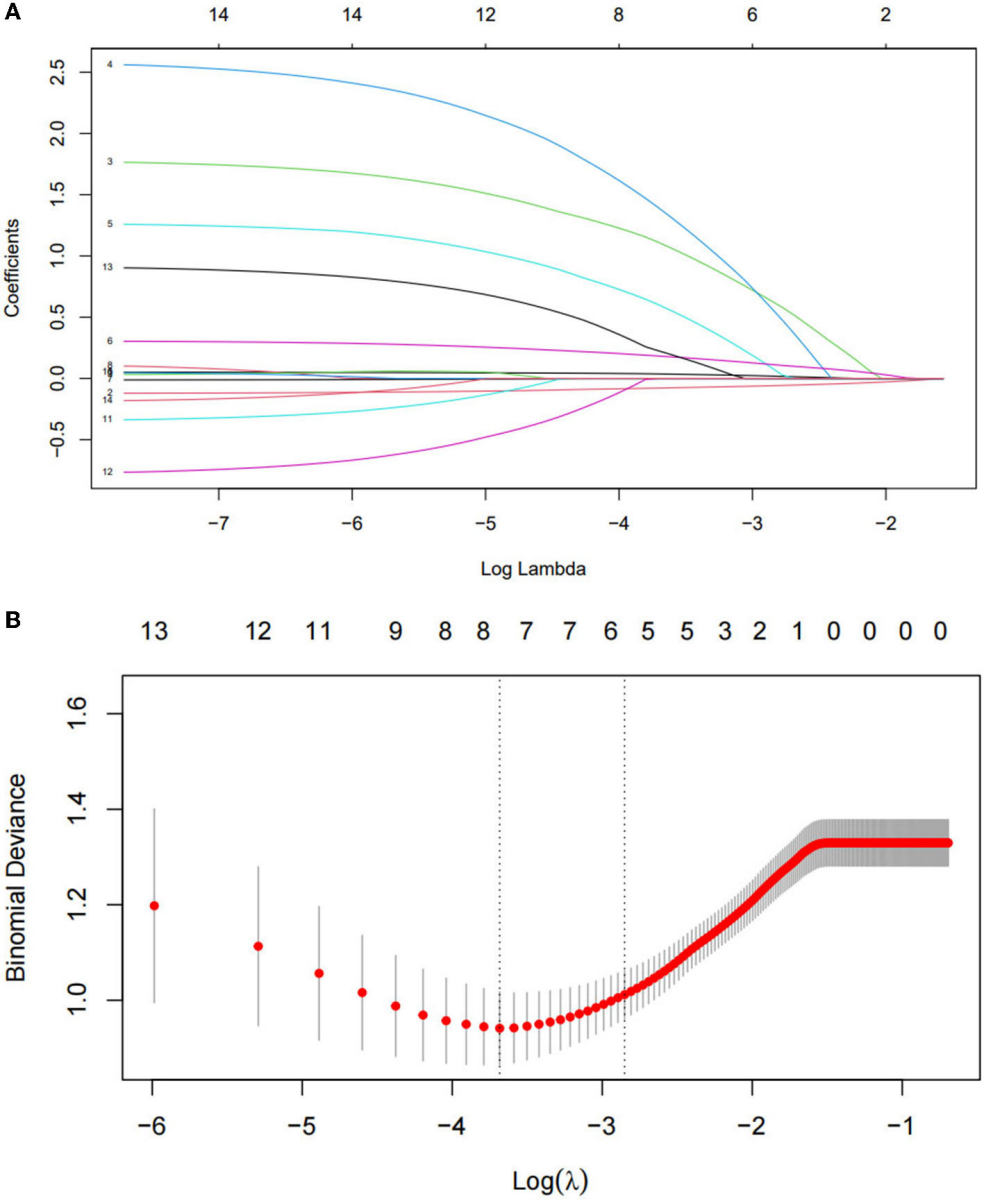
Optimal parameter (lambda) selection in the least absolute shrinkage and selection operator (LASSO) model **(A)**. Identification of the optimal penalization coefficient in the LASSO model was achieved by 10-fold cross-validation and the minimum criterion. The left vertical line represents the mean-squared error, and the right vertical line represents the cross-validated mean-squared error within 1 standard error of the minimum **(B)**.

All 14 variables were included in the LASSO regression for analysis, and the lambda (λ) parameter was chosen according to the minimum criterion in the LASSO model, using 10-fold cross-validation (Figures 2A,B). To provide a simple and accurate clinical model, six variables corresponding to the minimum mean square error log (λ): age, family history of constipation, total ALSFRS-R score, site of onset, total PSQI score, and depressed, were selected for inclusion in the model.

### Multivariate logistic regression analysis of the variables selected by LASSO

The results of multivariate logistic regression analysis of the variables selected by LASSO are shown in Table 2. The following variables were included in the model: age OR = 1.062 (95% CI: 1.010–1.118), family history of constipation OR = 13.717 (95% CI: 1.267–148.516), total ALSFRS–R score OR = 0.897 (95% CI: 0.844–0.953), site of onset OR = 3.346 (95% CI: 1.050–10.657), total PSQI score OR = 1.339 (95% CI: 1.112–1.612), and depressed OR = 5.792 (95% CI: 1.044–32.136).

**Table 2:** Logistic regression analysis of risk factors for constipation in ALS patients.

	**Group**	***B***	**SE**	**Wald values**	***P*-value**	**Odds ratio (95% CI)**
Age		0.061	0.026	5.411	0.020	1.062 (1.010–1.118)
Family history of constipation	No					
	Yes	2.619	1.215	4.643	0.031	13.717 (1.267–148.516)
ALSFRS-R total score		−0.108	0.031	12.219	< 0.001	0.897 (0.844–0.953)
Site of onset	Bulbar					
	Limb	1.208	0.591	4.175	0.041	3.346 (1.050–10.657)
PSQI total score		0.292	0.095	9.487	0.002	1.339 (1.112–1.612)
Depressed	No					
	Yes	1.757	0.874	4.037	0.045	5.792 (1.044–32.136)

### Construction and validation of nomogram risk model for predicting constipation in ALS patients

**Figure 3:**
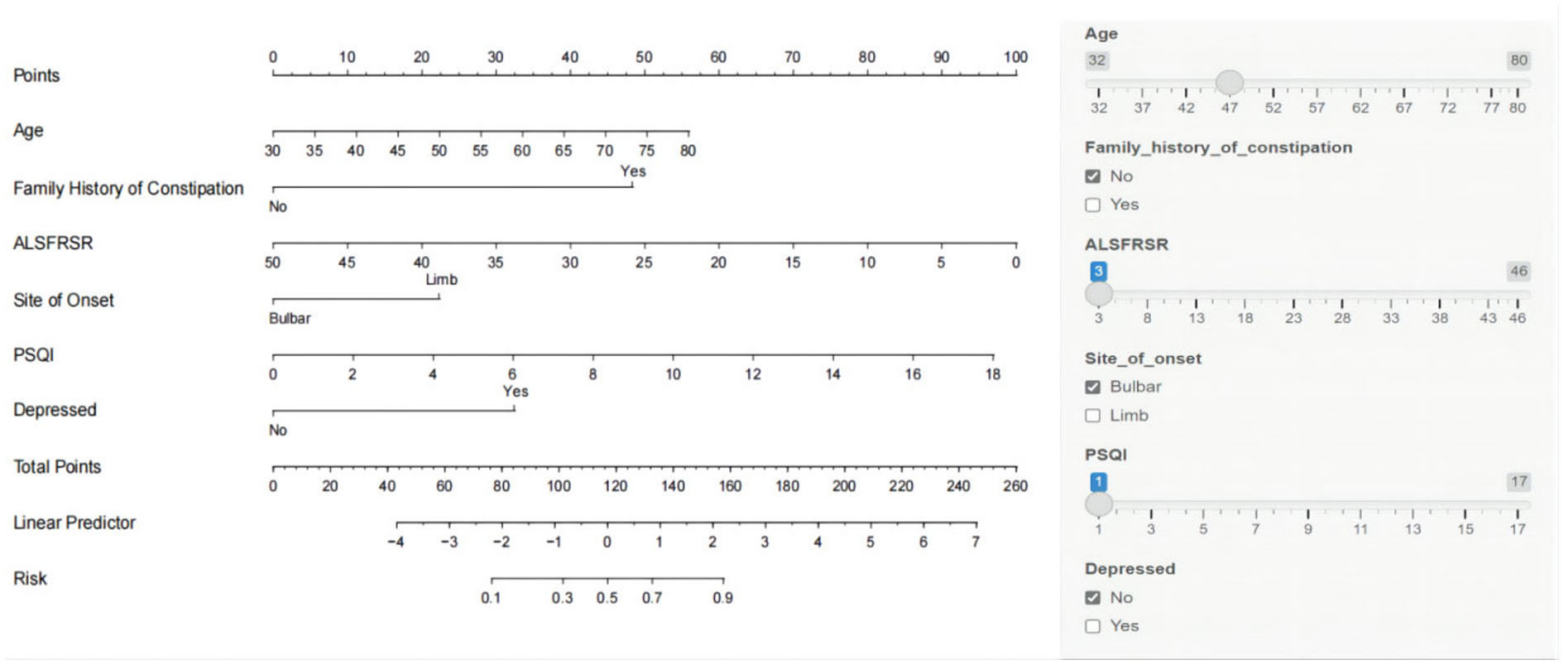
Nomogram model for predicting the risk of constipation in patients with amyotrophic lateral sclerosis (ALS).

A nomogram was constructed based on age, family history of constipation, total ALSFRS-R score, site of onset, total PSQI score, and depressed (Figure 3). The total patient score was calculated from the top score corresponding to the value of each predictor variable, and then the total score was used to read off the corresponding probability of constipation risk. We also built a web-based calculator (https://n18932925939.shinyapps.io/TongYangNiu/) to promote the use of the nomogram by clinicians. The predicted probability of constipation in ALS patients can be easily obtained after entering clinical variables and reading the output generated by the website.

**Figure 4:**
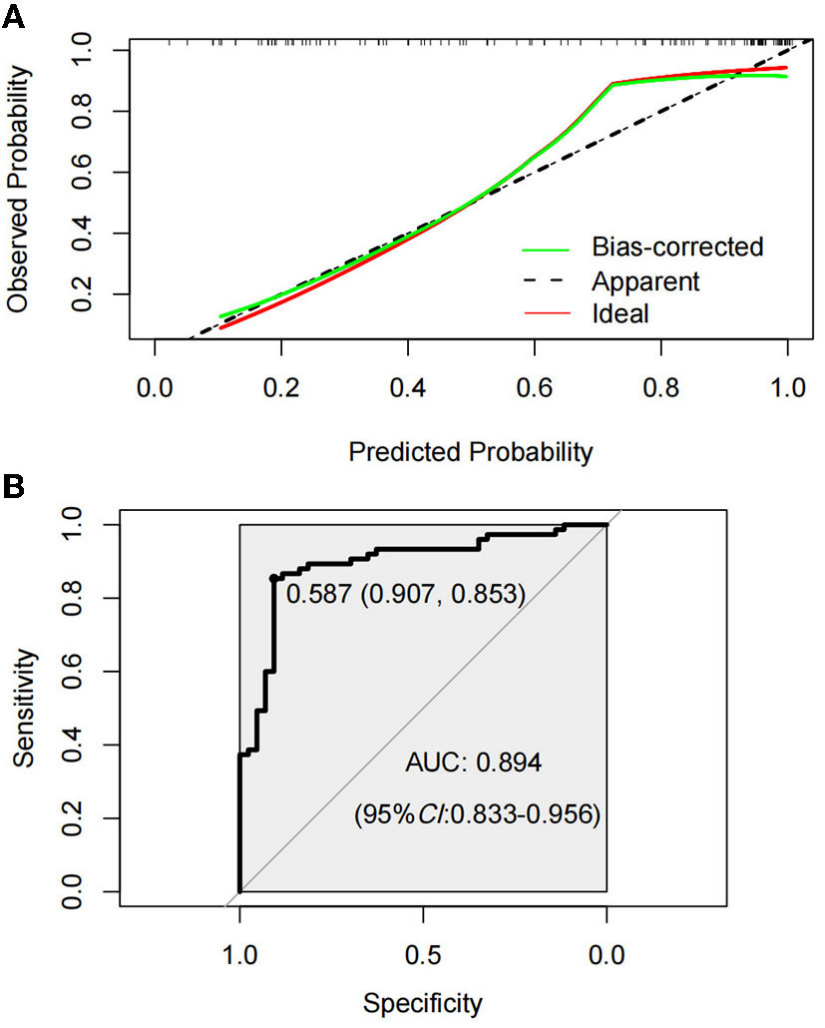
Calibration curve of amyotrophic lateral sclerosis (ALS) constipation model prediction **(A)**. Receiver operating characterization (ROC) curve of the model for predicting constipation in ALS patients **(B)**.

**Figure 5:**
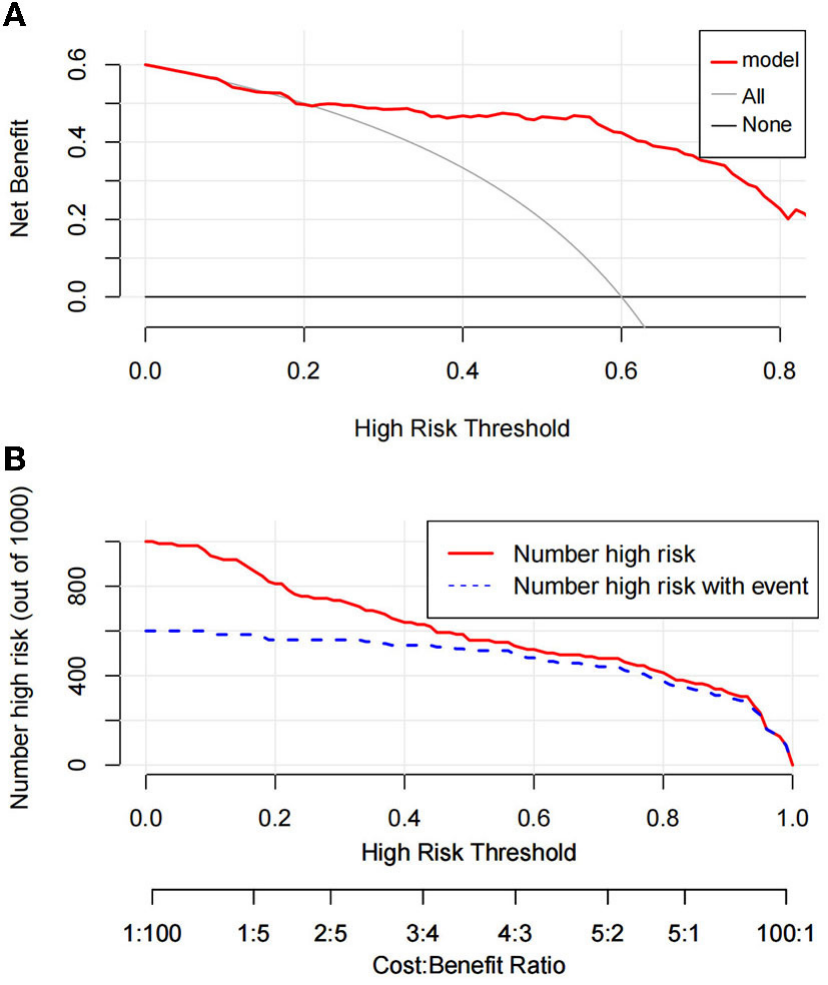
Decision curve analysis of prediction model **(A)**. Clinical impact curve of prediction model **(B)**.

The Hosmer–Lemeshow test was used to test the level of prediction bias (1,000 repetitions of the original data using the bootstrap method). This yielded χ^2^ = 11.11 (*P* = 0.195), suggesting that the model had good prediction accuracy (Figure 4A). The Brier score was 0.121, AUC was 0.894 (95% CI: 0.833–0.956), and C-index was 0.871 after 1,000 replicates using the bootstrap method, suggesting that the model had good discrimination (Figure 4B). The cutoff value of the model was 0.587 and the Youden index was 0.76, corresponding to a sensitivity and specificity of 85.3% and 90.7%, respectively. Figure 5A shows the results of the DCA of the prediction model. At the optimal cutoff value of the model in the ROC analysis (58.7%), the decision curve of the model lies above the None and All lines, and the model has clinical utility. The CIC (Figure 5B) shows that the number of positive cases predicted by the model was close to the actual number of positive cases. As the risk threshold increases, the number of cases predicted by the model became closer to the actual number of cases. Thus, the DCA and CIC further imply that the nomogram showed a better net benefit over a wide range of threshold probabilities and influenced patient prognosis.

### Spearman correlation analysis

**Figure 6:**
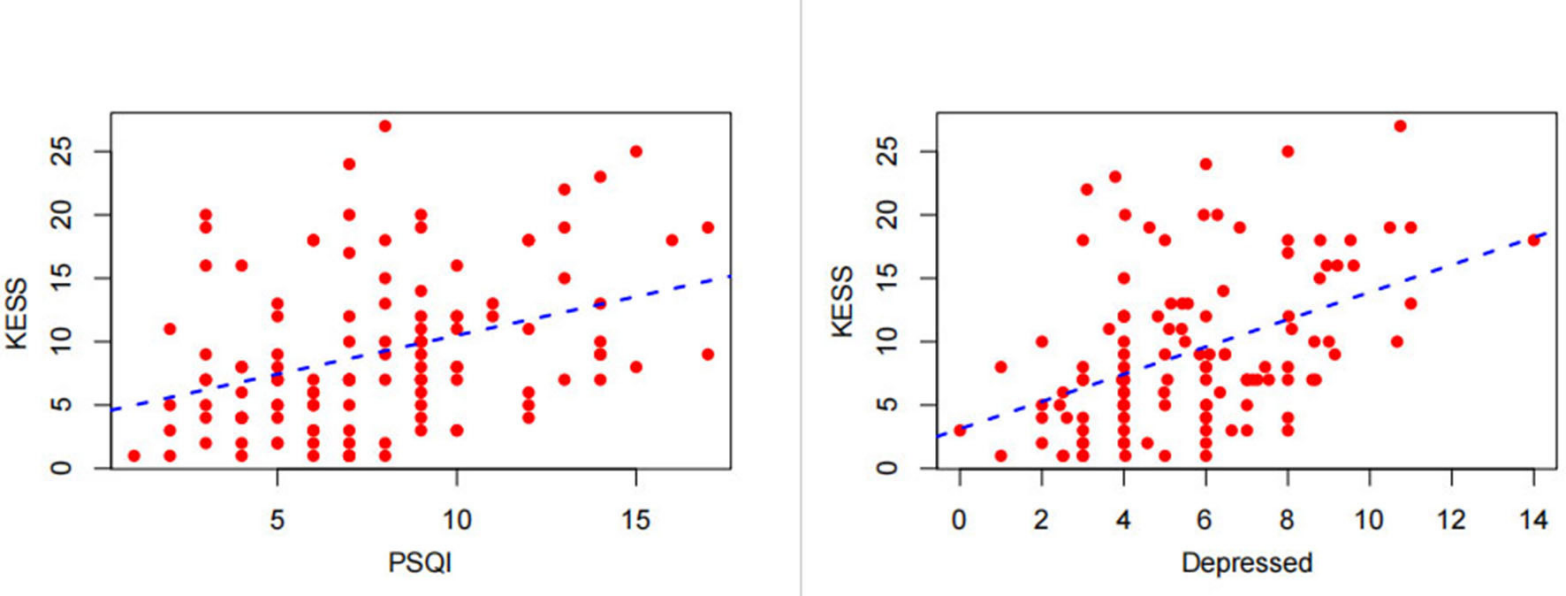
Correlation of the total Knowles–Eccersley–Scott Symptom Questionnaire (KESS) score with the total Pittsburgh Sleep Quality Index (PSQI) score and Hospital Anxiety and Depression Scale (HADS)-depression score.

Spearman's correlation analysis was performed to analyze the relationship between total KESS score and total PSQI score and depressed in all ALS patients. We observed a positive correlation between KESS and the variables, and an increase in total KESS score was associated with higher PSQI scores (Spearman's *r* = 0.377, *P* < 0.001), and depression scores (Spearman's *r* = 0.429, *P* < 0.001), as shown in Figure 6.

## Discussion

Despite being a common non-motor symptom in ALS patients, both neurologists and ALS patients have a low level of awareness of constipation, leading to frequent missed diagnosis of constipation symptoms by clinicians, which increases patients' suffering and hampers care of patients' families. We found that constipation occurred in 75 of 118 patients, yielding an incidence of 63.6%, which is much higher than that in the general population. We then developed and validated a simple and practical nomogram for predicting the risk of constipation in ALS patients, which has not been presented previously. The prediction model included age, family history of constipation, the ALSFRS-R total score, site of onset, PSQI total score, and depressed. It is an objective and simple tool for screening patients at high risk of constipation, with appropriate predictors entered in a dynamic online manner while the ALS patient is hospitalized, which simplifies the screening process and facilitates clinician decision-making.

This study applied LASSO regression analysis, which performed well in reducing the dimensionality of data in terms of variables and reduced multicollinearity, and then selected variables for multivariate logistic regression by minimizing coefficients and reducing variance. Variables were selected by 10-fold cross-validation, after which a logistic regression model was used to create a nomogram. The predictive model was internally validated, with *P* > 0.05 in the Hosmer–Lemeshow test, indicating a good fit of the nomogram. The initial AUC was 0.894, and after using 1,000 bootstrap resampling, the C-index was 0.871, which indicated that the model had excellent discriminatory power. The Brier score of 0.121 and the calibration curve indicated that the model had good calibration power. With a Youden index of 0.76, the corresponding sensitivity and specificity were optimal at 85.3% and 90.7%, respectively. In addition, DCA and the CIC indicated that the model was useful in clinical applications in terms of threshold probabilities.

This study revealed that age, a family history of constipation, the total ALSFRS-R score, site of onset, total PSQI score, and depressed influenced constipation in ALS patients. The clinical mechanism of chronic constipation is relatively complex, and its development is influenced by a variety of factors. Chronic constipation includes primary chronic constipation and secondary chronic constipation. The ALS patients in our study were patients with chronic primary constipation, which can be due to dietary factors (such as insufficient fiber intake), lifestyle factors (for example, lack of mobility or sedentary lifestyle), or a disorder of colonic propulsion or rectal emptying, colonic fecal motility regulation dysfunction, anorectal neuromuscular organ incoordination, and bidirectional bowel dysfunction due to brain–gut axis dysfunction (15, 16).

### Uncontrollable factors associated with constipation: Age, family history of constipation, ALSFRS-R Score, and limb onset

In ALS patients, constipation is influenced by age and family history of constipation. The reason why age affects constipation in ALS may be that the number of neurons in the myenteric plexus decreases with age, and the response to direct stimulation is impaired, which ultimately leads to the dysfunction of the myenteric plexus. Or the amplitude of inhibitory nerve input in the muscular layer of the colon circulation decreased, resulting in a lack of segmental motor coordination (28). Or the common factors that may cause constipation in the elderly include changes in diet habits, such as low dietary fiber content, high protein and fat content, or movement disorders, which increase the risk of constipation (17). On the other hand, constipation of ALS patients is also affected by family history of constipation. There are relevant literature reports that constipation patients with positive family history have obvious clinical characteristics compared with sporadic cases, that is, early onset age, long duration of constipation symptoms, fewer predisposing factors, more complications, and high frequency of stool medication use. The author believes that genetic factors may be the main factor in constipation population (29).

But our study also found that the ALSFRS-R score also influences the occurrence of constipation in ALS patients. The possible reasons for this are that the ALSFRS-R score mostly reflects the motor function of the brainstem and spinal cord in ALS patients. If the disease starts in the bulbar region, patients have difficulty swallowing and have relatively insufficient fluid and dietary fiber intake (30, 31), whereas, if the onset occurs in the limbs, the patient may have abdominal muscle involvement, resulting in constipation due to the inability to use thoracic and abdominal muscle strength during defecation, or because the patient is bedridden for a long time, resulting in long colonic transit time (32). Both insufficient drinking water and reduced activity of ALS patients can lead to constipation (5, 33). Patients with bulbar onset are prone to insufficient drinking water, while patients with limb onset are prone to decreased daily activity. Scagnelli et al. (31) found that bulbar-onset patients are more likely to occur inadequate intake of total body water and water turnover than limb-onset ones, so as to be easier to suffer from constipation if not considering the risk factor of muscles weakness. But interestingly, our study found that patients with limb onset were more likely to have constipation than those with bulbar onset. Therefore, we speculate that limb onset has a greater impact on constipation.

### Controllable factors associated with constipation: Depression and sleep disorders

Sleep disturbance and depression can be both a trigger for the development of constipation and a consequence of constipation. Therefore, we analyzed the correlation between the severity of constipation and depression and sleep disorders in our patients and found that they were strongly correlated. It is increasingly thought that communication within the microbiota–gut–brain axis functions bidirectionally. This characterization of the microbiota–gut–brain axis has crucial implications for the understanding and treatment of constipation and associated psychiatric disturbances (34). This feature has crucial implications for the relationship between constipation and depression. Constipation and depression interact with each other, as patients with constipation often have accompanying depression (35), and depression can likewise trigger constipation. It has been shown that probiotics have potential antidepressant-like activity in a constipation-induced depression animal model, and this effect may protect neuronal health by activating the AKT signaling pathway, thereby alleviating constipation-induced depression (36). Probiotics can improve sleep disorders in patients and can improve constipation symptoms, but no studies to date have investigated whether probiotics can improve constipation and thereby sleep disorders. In addition, our study found that up to 28.0% of patients with ALS had long-term problems with promoting defecation by enemas, such as a glycerin enema, which can resolve the problem of constipation for patients in the short-term, but which reduces bowel sensitivity with long-term application and can lead to paradoxical contraction of the anorectal ring (predominantly the puborectalis muscle) during forceful defecation, aggravating the symptoms of constipation (37). Therefore, for ALS patients predicted to be at high risk of constipation, early prophylactic administration of probiotics can be attempted, which can regulate intestinal flora, thus improving constipation, sleep disorders, and depressive states derived from constipation, and which may act as a preventive treatment for patients with ALS with constipation (38).

The dynamic web-based nomogram (https://n18932925939.shinyapps.io/TongYangNiu/) application developed in this study can be used to calculate the risk probability of constipation in ALS patients. This tool will facilitate early interventional treatment of constipation, including improving lifestyle, establishing good bowel habits, adjusting the patient's psychosocial status, and applying laxative medications, such as probiotics. It will also help neurologists to make better clinical decisions for these patients.

This study has some limitations. First, this was a prospective study and was prone to bias in patient selection, data collection, and analysis. Second, the missing data were processed by multiple imputation which may reduce the accuracy of the model. Moreover, it was a single-center study with a small sample size, which limits the generalization of the results; hence, further multicenter external validation is needed to verify the discriminating ability and generalizability of our nomogram. Risk of bias assessment is an essential step in any predictive modeling study. The Prediction Model Risk of Bias Assessment Tool (PROBAST) score (39) is high for this article because it has no external validation.

In summary, this study developed and validated an online nomogram based on independent risk factors to predict the probability of constipation risk in patients with ALS dynamically. This study provides a basis for clinical identification of ALS patients at high risk of constipation, which can allow early treatment with bowel function interventions.

## Data availability statement

The raw data supporting the conclusions of this article will be made available by the authors, without undue reservation.

## Author contributions

TN, HD, and YL designed the study and drafted the manuscript. TN, XZ, XL, and TL contribute to data collection. TN wrote the paper. QL and RL supervised the study. All authors have read and approved the manuscript for publication.
